# Addition to Staff of Buffalo Medical Journal

**Published:** 1912-12

**Authors:** 


### Addition to Staff of Buffalo Medical Journal

Dr. C. A. Ewald of Berlin, has accepted the position of Associate Editor for Germany and promises soon to contribute an article, probably on diseases of the oesophagus. Dr. Ewald is well known to the physicians of western New York, not only on account of his pioneer treatise on Diseases of the Stomach, which became the standard for subsequent books by many authors, but in a personal way, since he was the guest of the Buffalo Academy of Medicine a few years ago, at what was, at the time, the largest meeting in the history of the Academy. Dr. Ewald's letter of acceptance is in excellent English which, indeed, he speaks and writes more fluently than many American Gastro-enterologists. We extend to Dr. Ewald, a most cordial welcome which, we feel sure, our readers will second.

